# Five Steps Towards Avoiding Narrative Traps in Decision-Making

**DOI:** 10.3389/fpsyg.2021.694032

**Published:** 2021-08-12

**Authors:** Paul Dolan, Amanda Henwood

**Affiliations:** London School of Economics and Political Science, London, United Kingdom

**Keywords:** narratives, biases, heuristics, COVID-19, uncertainty

## Abstract

Narratives provide simple rules about how we ought to live and what our priorities ought to be. They are especially appealing in times of high uncertainty. Using the uncertainty surrounding Covid-19 as an illustration, we show how a narrative to preserve life has become dominant, and we illustrate how it has been reinforced by several behavioural biases. We argue that being able to identify and critically evaluate the impact of dominant narratives is vital to ensuring optimal decision-making. To facilitate this, we offer five recommendations—the ABCDE of decision-making—that can help to reduce the “narrative trap” in decision-making in any uncertain environment.

## Introduction—The Power of the Story

Dominant social narratives are stories told by society about the way things ought to be. They are communicated through social and cultural institutions, by mass media, and via social networks (Rappaport, 2000). Social narratives have been shown to powerfully shape decision-making in many domains, including marketing (Escalas, 2007), economics (Shiller, 2017), and politics (Merkus et al., 2014). In an example of how stories influence health-related behaviour more than evidence, one study altered text highlighting the risks of sunbed use. The “narrative” condition detailed the story of a young woman's experience using sunbeds and getting skin cancer and the “statistics” condition simply presented facts about sunbeds and their relationship to skin cancer. Those who received the narrative text reported much higher intentions to engage in skin protecting behaviours, such as not tanning and wearing sunscreen, than those in the statistical text group (Greene and Brinn, 2003).

The personal and social stories that become dominant are heavily influenced by the values, desires, and life experiences of decision-makers. Narratives form part of a larger sense-making process that helps us to ascribe meaning to our lives (McAdams and McLean, 2013). Shaped by our interactions, social narratives become the means through which we are able to assert our values and identities. Socio-cultural models propose that narrative identity builds over time as individuals engage in a shared sense-making process with others (McLean et al., 2007). The close tie with our identities is one significant reason why social narratives have become so ingrained in so many of the decisions we make for ourselves and on behalf of others (Sewell, 1992). Narratives also tend to originate from the most powerful individuals and groups in society, thus affording extra weight to the values, desires and life experiences of powerful people (Hampton, 2009).

In defining narratives, some scholars place emphasis on progression toward individual goals over time (Cialdini and Goldstein, 2004), whilst others consider particularly salient ideas as signals of dominant cultural narratives (Shiller, 2017; Tubadji et al., 2020). We are not concerned with the variations here but rather with the general idea that, building upon an underlying set of values, certain stories become dominant social narratives adopted by society. In this sense, narratives are akin to social norms, which are comprised of behavioural regularities that individuals will be judged harshly for deviating from Hechter and Opp (2005).

Whilst the reassurance and sense-making that narratives provide is certainly beneficial (McAdams, 2006), following them blindly poses a signficant threat to decision-making (Dolan, 2019). The narrative that “people should strive to earn as much money as they can,” for example, suggests that we should take jobs with higher salaries whenever the opportunity arises. This might serve us well when we are poor but the narrative may lead us to take jobs without proper regard for our work-life balance and other factors that are critical to our well-being (Kahneman and Deaton, 2010). Therefore, being alert to dominant social narratives that guide our decisions, and to the point at which they may yield potentially negative effects, is essential for promoting individual and social welfare.

Social narratives will most powerfully influence decisions under conditions of uncertainty. There is good evidence that we become more reliant on simple rules of thumb when decision outcomes are uncertain (Mousavi and Gigerenzer, 2014) and social narratives provide us with simple guides to decision-making. These decision-making short cuts, also known as heuristics, certainly help make decisions easier, but their effects on the consequences of those decisions is ambivalent. On the one hand, they can help us to ignore spurious reasons to act in a particular way and to instead focus on those aspects of the decision problem that are most likely to lead to the best oucomes (Mousavi and Gigerenzer, 2017). On the other hand, they can misdirect us from properly accounting for the full costs and benefits of our decisions and to instead focus only on a limited subset of relevant factors (Simon, 1997). Although there are a number of heuristics that impact upon the decision-making process, the degree to which social narratives help or hinder effective decision-making has received very little attention.

In this paper, we consider the potential for dominant social narratives to *influence* the decision-making process. We stress the importance of paying attention to their impact in order to establish whether, on balance, narratives help or hinder effective decision-making. We contend that the failure to step back and consider the impact of narratives will impede effective decision-making by leaving the decision-maker open to unspecified and unrecognised bias. Narratives are not good or bad in themselves, but their ability to make some decisions appear more appealing than others—often in ways that lie below our conscious awareness—is detrimental to effective decision-making.

Given the uncertainty surrounding COVID-19, we focus on the public policy decisions in response to the pandemic, which around the world have primarily sought to suppress the virus. We illustrate how an already dominant social narrative—namely, “preserve life”—will have increased the attractiveness of certain policy decisions over others. We show how other important aspects that bias behaviour (social norms, fear, and situational blindness) can increase the power of dominant narratives in decision-making. We do not comment on the appropriateness of the policy decisions discussed in our examples, but rather highlight the extent to which our evaluations of them may be influenced by the power of the preserve life social narrative.

In the final section of the paper, we provide the first framework to help policy-makers and other decision-makers better attend to the possible influence of narratives on their decisions. The five steps we set out—the ABCDE of decision-making—are intended to be useful in any decision-making environment, and not just in response to a crisis such as COVID-19. Indeed, since social narratives can impact upon any decision-making environment, we recommend that the framework is used widely. We provide examples of other decision-making contexts where social narratives may have a significant impact.

### The Preserve Life Narrative

Across the world, a natural inclination to preserve life and fight death right up until the bitter end is widespread. The preserve life narrative prescribes that we should all prolong our lives in good health for as long as possible even when we incur significant financial and well-being costs in doing so. In a clear example of this, not only do healthcare costs in the last year of life account for about one-quarter of total healthcare expenditure (Duncan et al., 2019), but higher levels of death anxiety are associated with a host of negative psychological effects (Coupland, 2020). In contrast, terminally ill patients who know about and accept their prognosis experience less mental suffering in the time leading up to death. We cannot claim that spending a quarter of the health budget in the last year of life is an inefficient use of resources, but it is entirely possible that the preserve life narrative fuels some of this spending.

Further evidence of the preserve life narrative can perhaps be found in our unwillingness to legislate in favour of assisted dying in the UK and many other parts of the world. The current law on assisted dying in the UK states that there are no circumstances under which people can receive medical assistance to end their lives, even with strict safeguards in place. Again, we cannot assert that the narrative to preserve life does not also increase social welfare by acting as a barrier against vulnerabe people being coerced into ending their lives but many people go through significant mental and physical suffering in their last years of life and around 300 terminally ill people take their lives in England each year (The Sunday Times, 2021). So, any benefits of the current law need to be set against these costs.

Terror Management Theory, which posits that the fear of death drives much of human behaviour, helps to explain a lot what is going on here (Greenberg et al., 1992). According to this theory, our fear of death leads us to employ two protective mechanisms to help us cope with this fear. First, we endorse certain worldviews such as a belief in an afterlife, which gives us a sense of “symbolic immortality.” Second, we bolster our self-esteem, by viewing ourselves as an essential part of the culture within which we are embedded. An impressive body of work comprising nearly 300 experiments has shown that investment in cultural worldviews and self-esteem buffers the potential for death anxiety under conditions of increased mortality salience (Burke et al., 2010). This natural inclination toward self-preservation likely fuels our affinity for the preserve life narrative, even under circumstances where our values conflict with the narrative (such as believing in the right to self-determination relating to assisted death). Under conditions of heightened fear and mortality salience, the power of the preserve life narrative may become especially strong.

Arguably, another underlying value in society is that human life is a protected or sacred value (Waldmann et al., 2012). This standpoint can function as an important foundation for social justice, since it encourages us to argue against political practises that threaten human life and cause widespread suffering. Yet on the other hand, it has made the possibility of trading off human life for other attributes of value, such as income or mental health, appear impossible, and immoral (Singer, 2016). Those who do seek to compare the value of a human life against other values evoke a feeling of moral disgust in others and risk social disapproval (Tetlock, 2000). We certainly do not rule out the possibility that other factors are at play, but it is likely that fear and deeply held societal values about the sanctity of life increase our affinity for the preserve life narrative. These values might not always be aligned with increasing social welfare.

When COVID-19 hit, governments were faced with the difficult decision of whether, and by how much, to prioritise policies seeking to suppress the virus over those seeking to mitigate its harms, whilst allowing for more economic activity and social interaction (Pearce et al., 2020; Wang and Flessa, 2020). Across the world, the preserve life narrative functioned as a direct call to action, and the effects of the virus for mortality risks came to dominate public communication and policy responses (Ogbodo et al., 2020). “Stay home, save lives, protect the NHS” was the dominant government messaging at the advent of the pandemic, which had a very clear health framing. Discourse analysis of the UK media coverage during the pandemic has highlighted three main narratives, each of which speak to the larger preserve life narrative we discuss: “(*1) fear of an uncontrollable, unknown new virus and its uncertain consequences—associated with sensationalist language…(2) managing uncertainty and fear via calls for behaviour change, associated with use of war metaphors; and (3) mourning and loss narratives*” (Sowden et al., 2021).

In the UK, the response to COVID-19 was repeatedly said by the Government to have been guided by “the science.” But the science can never be clear-cut and is much easier to “sell” if it is underpinned by a clear narrative. In an example of this narrative persuasion, key figures such the UK Secretary of State for Health, Matt Hancock, used fear-evoking, militant terminology in describing the UK's planned approach to tacklng Covid-19: “We are at war against an invisible killer and we have to do everything we can to stop it” (Denham, 2021). Here saving lives was highlighted as the ultimate, and only, end goal. A focus on communicating and enacting policies to stop death and bereavement was clear. This kind of coverage provided a dominant lens through which the public were encouraged to view the crisis. Arguably, this resulted in less attention being paid to education, mental health, and income related decision outcomes, which also pose a signficiant and direct, but less immediate, threat to human life.

In one way or another, all governments would seek to suppress virus transmission rates to reduce mortality risks, and so the question becomes by how much and at what cost. We know that people regularly make trade-offs between various attributes of value to their lives. Even amid a lockdown, many people would willingly put their own health at risk to be able to see their grandchildren, or to attend the funerals of loved ones. Similarly, citizens and policy-makers care about mortality risks (ONS, 2011), but also care about mental health (Department of Health, 2014), and education (Britton et al., 2019). There will therefore come a point at which the costs of restrictions become too high a price to pay for the reduction in mortality risks. Consequently, we must be able to appraise whether accepting higher rates of virus transmission and associated increases in mortality risks from COVID-19 might lead to greater social welfare when all the costs and benefits of the various policy options are properly accounted for.

## Behavioural Biases That Endorsed a Preserve Life Narrative

Narratives can be enhanced by existing behavioural biases. We must be alert to these biases when trying to mitigate the influence of narratives on decision-making. Below, we highlight three behavioural biases that reinforce many social narratives: following the first, fear and situational blindness. We provide examples of how these factors have accelerated the take up of the preserve life narrative in response to COVID-19. To reiterate, we do not seek to make any substantive claims about the rightness of different decisions, only to show how the preserve life narrative will have been magnified by these effects.

### Following the First

Much of our own behaviour is heavily influenced by what other people are seen to do first. There is a wealth of psychological and behavioural evidence highlighting our heavy reliance on other people for cues on how to behave (Milgram et al., 1969; Cialdini and Goldstein, 2004; Campbell-Meiklejohn et al., 2010). When we are uncertain about what to do, we are even quicker look to the behaviour of others to guide us (Smith, 2007). Whoever responded first to COVID-19 was going to set the tone for the nations that followed. Consider China's response to COVID-19, which was to lock down Wuhan. When the UK went into a national lockdown on 23 March 2020, it was after Italy, Spain, France and Ireland had imposed quite stringent restrictions on personal freedoms. Lockdown might still have been the best policy in the UK by preventing the healthcare system from becoming overwhelmed, but lockdowns elsewhere that were justified on this basis will have made it harder for proper consideration of the wider health, economic and social impacts.

### Fed by Fear

Our desire to avoid negative emotions is much stronger than our desire to approach positive ones (Baumeister et al., 2001), and so narratives that target and seek to mitigate fear are very appealing. When choosing between policies about infection control, suppression policies are generally preferred to mitigation ones because they are aligned with narratives that help us to deal more directly with our fears of the virus. Suppression policies might still be more effective than mitigation policies, of course, but not for this reason. One study explored the impact of national lockdowns on national anxiety levels by using the frequency of “Death” as a search term in Google, and found that national levels of anxiety rose steeply in the UK when Italy went into lockdown (Tubadji et al., 2020). Anxiety fell a little once the UK went into lockdown, suggesting that some of the initial anxiety was due to the differences in policy between the two countries.

It is of course rational in a substantive sense to be afraid of a new virus with relatively high mortality risks and a pretty grim process of dying. And it also makes sense for governments to play on our fears to some extent in order to get us to comply with the restrictions they put in place (Van Bavel et al., 2020). Fear motivates us to avoid harm (Michie et al., 2020). Indeed, increasing the “perceived level of personal threat” in the public was a listed an evidence-based strategy for increasing adherence to social distancing policies by government advisors (Scientific Advisory Group for Emergencies, 2020). But making younger people scared of dying in ways that are wholly disproportionate to their own risks is very different from making them concerned about the effects of their behaviour for the risks of older people, and raises serious ethical concerns about how the government and the media has deliberately evoked fear in people that may be difficult to reverse (Henwood, 2020).

There have been many news reports on the absolute number of deaths from Covid-19 without the figures ever being placed in context—for example, as a percentage of mortality by all causes. This has promoted hypervigilance to COVID-19 deaths (Cole et al., 2013), which stands in the way of us adapting to our fears through an understanding of the actual levels of risk (Haegler et al., 2010). We know from risk perception studies that the more aware of risks people become, the more fearful of them they will be (Fox, 2006). Studies also show that under conditions of uncertainty people place a larger weight on small probabilities if they are fear-inducing (for example, the probability of a car crash) (Rottenstreich and Hsee, 2001). Overall, the exacerbation of fear succeeds in pushing the preserve life narrative.

### Spurred on by Situational Blindness

The famous “invisible gorilla” experiments powerfully illustrate that when you attend to one aspect of your environment you do not attend to another (Hama, 2010). This can lead to situational blindness, whereby you are so focused on one aspect that you fail to notice the bigger picture. When COVID-19 arrived, it was obvious that most attention would be directed at the mortality risks from the virus. But this also means that policymakers did not pay enough attention to the invisible gorilla of harms caused elsewhere. As one example, the message to “Stay at home. Protect the NHS. Save Lives” resulted in people not going to hospital with serious health problems, such as suspected cancers, which could eventually result in more life years being lost than from COVID-19 (Maringe et al., 2020).

Some of this situational blindness could be explained by a natural human proclivity to focus on immediate effects over delayed ones. The deaths from COVID-19 are now, those from cancer will be later, and today matters more than tomorrow. But this is only part of the story. When governments around the world decided to close schools, they were forcing many children to stay at home in dysfunctional households and away from a school environment, which may have been the only place they received a decent meal, let alone care. Governments were harming many children with immediate effect and so it is not only the immediacy of COVID-19 deaths that made them more salient.

Perhaps the biggest differences with mortality risks compared to misery impacts is their permanence and the related ease with which they can be attributed to policy responses. There is no recovering from death and whilst deaths can be attributed more directly to policy responses, other impacts, such as mental health, are harder to prove as having a direct relationship to policy given the many other factors by which they can be influenced. Moreover, whilst many children's lives will be significantly, and perhaps even irreparably, damaged from missing many months of school, their lives are not literally over, and there is always the small glimmer of hope that their lives can be turned around. Perhaps this is all how it should be, but situational blindness directs us toward further downplaying psychological impacts that could well turn out to be irreversible (Mental Health Foundation, 2020).

## Actionable Recommendations For Avoiding the Narrative Trap in Decision-Making

Using COVID-19 as an example, we have highlighted the potential for narratives to enhance the attractiveness of certain decisions over others, especially in the context of radical uncertainty. The ABCDE of decision-making set out below will help us to rebalance the impact of dominant social narratives in ways that allow for an assessment of whether such narratives are having a welfare enhancing impact on the decision-making process. These simple rules can be applied to any decision-making environment, and are summarised in the table below:

**Table:** 

**A**cceptance	Accept the potential influence of narratives on decision-making
**B**alancing	Identify the dominant narratives and balance them against other stories
**C**hecklists	Create a checklist of the different impacts any given decision will result in
**D**iversity	Include people with a diverse range of backgrounds and perspectives
**E**valuation	Quantify and compare the effects across different domains

The first step is acceptance. To understand why we act and feel in the ways we do, and to change those behaviours and emotions, we must first accept who we are and our actions, thoughts and feelings (Hayes et al., 2012). To understand the role that narratives play in determining our decisions, we must accept their potential influence on our behaviour. Greater acceptance of the emotions that drive us can also help us to manage negative emotional states such as anger and anxiety (Wolgast et al., 2011). In the context of COVID-19, we may well have been better able to assess the overall impact of different policies after accepting that our natural inclination will be to try and avoid deaths.

The second step is balancing. Stories are comforting, and so alternative stories are required if the dominant ones are to be challenged. We cannot simply rely on more and better evidence, because we can always find evidence to support a story we feel confident about (Rollwage et al., 2020). Preserve life has a simplicity and a strength to it in a way that something along the lines of “consider all the costs and benefits of action” does not. But “misery mitigation” might be a little more salient and can, in the very least, be considered alongside preserve life to show how different policies have different consequences for life expectancy on the one hand and life experiences on the other. Decision-makers should seek to first identify the dominant narratives and then think of some counter narratives. This enables us to maintain the sense-making that narratives provide whilst protecting ourselves from being unduly influenced by the power of one familiar narrative. Consider how the narrative “move fast and break things” might lead tech mogels to seek to disrupt too often and in contexts that do not demand it. Only after setting out competing narratives, can we properly consider how and in what ways narratives might be influencing how we are weighing the evidence and reaching our judgements.

The third step is checklists. Checklists work because they force us to evaluate our judgements in terms of pre-determined criteria, rather than just in terms of criteria we cherry-pick as being more important at that time. They are especially helpful in drawing us back from situational blindness. This is most frequently discussed in the aviation and medical sectors to describe the causes of errors made by pilots and surgeons when they miss crucial information in their environment (Haynes et al., 2009). For example, the World Health Organsation now routinely advise surgeons to use the Surgical Safety Checklist, which has 19 items across three critical time points: sign-in, timeout, and signout (World Health Organization and WHO Patient Safety, 2008). Completion of these critical steps ensures safer delivery of key medical services by reducing the likelihood of important omissions and improving situational awareness (Low et al., 2012). A COVID-19 checklist of impact might include attributes such as “mental health effects,” “effects on lifetime inequalities,” could be added to the impact checklist in addition to “mortality risks from COVID-19.” The decision-makers then have the responsibility to ensure that every decision made has considered each of these factors.

The fourth step is diversity. There is good evidence that performance is improved when a diversity of values and perspectives are involved in decision-making (Jackson et al., 995). Not only does diversity of values and perspective help to uncover neglected areas of impact from blindly following the dominant narratives, but it has also been proven to generate more creative solutions under conditions where perspective taking is encouraged (Jackson et al., 1995). We must seek to include decision-makers from as many disciplines, values, perspectives and experiences as possible. COVID-19 has been a health, economic, and social crisis, and yet health experts have dominated the discussions of how best to respond.

The fifth step is evaluation. There is the need to consider all the available evidence, and to evaluate what represents the best policy. An evaluation of policy responses should account for the full range of effects from an intervention. Attempts should be made to gather up the items on the checklist under (C) in ways that allow the various impacts to be compared to one another. Different indices could be used in different domains (e.g., aggregated health effects, aggregated income effects etc.). This allows policymakers to determine the weights attached to the different domains. Alternatively, and arguably ideally, all outcomes will be expressed in a single metric. Cost-benefit analysis (CBA), as recommended by the UK Treasury for example, seeks to express all costs and benefits in monetary units (The Green Book, 2013). For the purposes of cost-effectiveness analysis (CEA), a single metric of benefit is required but it does not have to be converted into monetary values. Evaluations of this kind and depth can help to address the impact of following the first, fear and situational blindness since they force us to scrutinise the choices made empirically. In the case of COVID-19, policymakers could be required to express the impacts of their policies in quality-adjusted life years (Dolan, 2001), for example, not just from COVID-19 risks but from the effects of missed cancer diagnoses, mental health effects and so on.

## Conclusion

We accept that combating long-standing dominant narratives is a difficult task, and it is naïve to believe that they can ever be eliminated completely from any decision-making process. Indeed, since they can sometimes be helpful—particularly when it comes to sense-making—they shouldn't be removed entirely. But the five steps set out here can help to reduce the power of the most dominant stories and avoid the potentially large-scale costs that may be incurred when we are *blindly* led by narratives about how the world ought to be. This is a call to action, then, for decision-makers everywhere to start paying more attention to the ways in which narratives can impact upon their decisions, especially in uncertain and high stakes environments.

## Data Availability Statement

The original contributions presented in the study are included in the article, further inquiries can be directed to the corresponding author/s.

## Author Contributions

All authors listed have made a substantial, direct and intellectual contribution to the work, and approved it for publication.

## Conflict of Interest

The authors declare that the research was conducted in the absence of any commercial or financial relationships that could be construed as a potential conflict of interest.

## Publisher's Note

All claims expressed in this article are solely those of the authors and do not necessarily represent those of their affiliated organizations, or those of the publisher, the editors and the reviewers. Any product that may be evaluated in this article, or claim that may be made by its manufacturer, is not guaranteed or endorsed by the publisher.
